# Upfront Systemic Chemotherapy and Short-Course Radiotherapy with Delayed Surgery for Locally Advanced Rectal Cancer with Distant Metastases: Outcomes, Compliance, and Favorable Prognostic Factors

**DOI:** 10.1371/journal.pone.0161475

**Published:** 2016-08-18

**Authors:** Hong In Yoon, Woong Sub Koom, Tae Hyung Kim, Joong Bae Ahn, Minkyu Jung, Tae Il Kim, Hoguen Kim, Sang Joon Shin, Nam Kyu Kim

**Affiliations:** 1 Department of Radiation Oncology, Yonsei Cancer Center, Yonsei University College of Medicine, Yonsei University Health System, Seoul, Korea; 2 Division of Medical Oncology, Department of Internal Medicine, Yonsei Cancer Center, Yonsei University College of Medicine, Yonsei University Health System, Seoul, Korea; 3 Division of Gastroenterology, Department of Internal Medicine, Yonsei University College of Medicine, Yonsei University Health System, Seoul, Korea; 4 Department of Pathology, Yonsei University College of Medicine, Yonsei University Health System, Seoul, Korea; 5 Department of Surgery, Division of Colorectal Surgery, Yonsei University College of Medicine, Yonsei University Health System, Seoul, Korea; University of Texas MD Anderson Cancer Center, UNITED STATES

## Abstract

**Purpose/Objective(s):**

Optimal treatment for locally advanced rectal cancer (LARC) with distant metastasis remains elusive. We aimed to evaluate upfront systemic chemotherapy and short-course radiotherapy (RT) followed by delayed surgery for such patients, and to identify favorable prognostic factors.

**Materials/Methods:**

We retrospectively reviewed 50 LARC patients (cT4 or cT3, <2 mm from the mesorectal fascia) with synchronous metastatic disease. The primary endpoint was progression-free survival (PFS). The secondary endpoints were overall survival, treatment-related toxicity, and compliance. We considered P values <0.05 significant.

**Results:**

At 22 months median follow-up, the median PFS time was 16 months and the 2-year PFS rate was 34.8%. Thirty-five patients who received radical surgery for primary and metastatic tumors were designated the curable group. Six patients with clinical complete response (ypCR) of metastases who underwent radical surgery for only the primary tumor were classified as potentially curable. Nine patients who received no radical surgery (3 received palliative surgery) were deemed the palliative group. The ypCR rate among surgery patients was 13.6%. PFS rates for the curable or potentially curable groups were significantly longer than that of the palliative group (P<0.001). On multivariate analysis, solitary organ metastasis and R0 status were independent prognostic factors for PFS.

**Conclusions:**

These findings demonstrated that a strong possibility that upfront chemotherapy and short-course RT with delayed surgery are an effective alternative treatment for LARC with potentially resectable distant metastasis, owing to achievement of pathologic down-staging, R0 resection, and favorable compliance and toxicity, despite the long treatment duration.

## Introduction

In locally advanced rectal cancer (LARC), mesorectal fascia (MRF) involvement is a significant prognostic factor influencing local recurrence and survival rates [1,2]. Preoperative long-course concurrent chemoradiation (CCRT) followed by TME is widely accepted as a standard treatment scheme for LARC [3,4]. A Dutch TME trial demonstrated that preoperative short-course radiotherapy (RT) followed by immediate surgery reduced local recurrence, but did not benefit patients with positive circumferential resection margins [5,6]. A Polish trial also demonstrated that conventional CCRT produced significantly more down-staging than short-course RT followed by immediate surgery despite no difference in survival and late toxicity [7]. Furthermore, down-staging has been recently reported after delayed surgery [8,9].

Currently, stage IV LARC with potentially resectable distant metastasis is considered to be distinct from stage IV disease with widespread distant metastasis. An effective treatment strategy for patients with the former disease remains elusive. Several studies about LARC plus synchronous distant metastases demonstrated that a curative approach including chemotherapy and short-course RT followed by delayed surgery could be an effective and feasible treatment [8,10]. Based on this, we conducted a phase II clinical trial (NCT01269229) [11], in which patients with LARC and synchronous liver metastases were treated with upfront systemic chemotherapy, short-course RT to the primary tumor and delayed surgery. The outcomes of this approach were described previously [12]. However, the clinical significance of this treatment scheme has not yet been established.

Hence, succceding our previous report [12], we retrospectively investigated our LARC patients outside of a clinical trial NCT01269229 (off-protocol) to determine oncologic outcomes and feasibility of the described therapeutic strategy. Furthermore, we aimed to identify the favorable prognostic factors in primary metastatic LARC cancer patients who were enrolled in this study.

## Materials and Methods

### Patient selection

This study was retrospective in design and received approval from the internal review board at severance hospital (IRB No. 4-2015-0076). Medical records of 82 patients with stage IV LARC with a limited number of metastatic lesions who underwent upfront chemotherapy and short-course RT with delayed surgery between 2009 and 2014 were reviewed. Among 82 patients, 32 enrolled in the prospective study [11]; the rest of patients did not participated due to refusal or ineligibility by lung, bone, or multiple organ metastases. These remaining 50 patients were reviewed retrospectively in this study. The patient records and information were anonymized and de-identified prior to analysis. Patients had primary rectal tumors with pelvic organ invasion (cT4) or mesorectal infiltration invasion with <2 mm distance from the MRF, and potentially resectable distant metastases. Patients were diagnosed with biopsy-confirmed adenocarcinoma as the primary rectal lesion. For the initial staging work up, digital rectal examination, sigmoidoscopy, pelvic computed tomography (CT), or MRI were performed to evaluate local tumor extent and the involvement of the MRF. Chest radiography, CT scanning of chest and abdomen, and positron emission tomography to identify distant metastasis were also performed. Patients had good performance statuses and normal pretreatment hematologic, renal, and hepatic functions.

### Multidisciplinary team approach

Assessments and treatment approaches were determined at a multidisciplinary team conference in the Colorectal Cancer Center at our institution as described previously [12]; candidates for upfront systemic chemotherapy and short-course RT with delayed surgery were selected with the intention of performing R0 resection for TME after tumor regression and simultaneous complete resection of metastatic lesions. Resectability of the rectal and metastatic lesions was assessed via imaging. All patients were administered 4 to 9 cycles (median 4 cycles) of upfront systemic chemotherapy with a FOLFOX (5-fluorouracil/leucovorin/oxaliplatin combination) or FOLFIRI (5-fluorouracil/leucovorin/irinotecan combination) based regimen with or without bevacizumab (Avastin) or cetuximab (Erbitux). RT of 25 Gy in five fractions was delivered for 5 consecutive work days 1 week following upfront chemotherapy. To allow for primary tumor regression, the same chemotherapy regimen (median 4 cycles, range 0–8 cycles) was performed between the end of RT and surgery for all patients except one. Chemotherapy was administered 1 week after RT. Surgical resection was performed after evaluation of resectability and treatment response at least 6 weeks after RT.

### Short-course radiotherapy

The target volumes and RT short-course technique were described previously [12]. We defined the gross tumor volume (GTV) as the primary tumor and any significant surrounding lymphadenopathy showing mesorectal infiltration. The clinical target volume (CTV) added a ≥2 cm margin to the GTV in superior and inferior directions, laterally encompassing the entire mesorectum at the level of the GTV. A 5-field technique (anteriorposterior, right-lateral, right-posterior-oblique, left-posterior-oblique, and left-lateral beams) covering the CTV with a 1 cm margin was utilized. The CTV was covered within the 95% isodose line of the prescribed dose.

### Tumor assessment, follow-up, response evaluation, and patterns of failure

We performed staging work-up before short-course RT and immediately afterwards, prior to surgery. The imaging studies were repeated to investigate the response and resectability. Patient follow-up was performed at 1, 3, 6, and 12 months, with imaging undergone at 1, 6, and 12 months after surgery, and annually thereafter. Treatment-related toxicities were assessed at every follow-up visit. We assessed acute treatment-related toxicities between RT and surgery. Post-surgical complications were also evaluated. Toxicity was graded based on the Common Toxicity Criteria, version 4.0, of the National Cancer Institute. We defined a complete response (CR) as a 100% decrease in gross tumor size on clinical or radiological evaluation. Partial response (PR) and progressive disease (PD) were defined as a ≥50% decrease or >25% increase in primary and metastatic gross tumors sizes or the detection of newly developed lesions, respectively. Otherwise, cases were categorized as stable disease (SD). We assessed pathologic primary tumor response using Mandard’s classification[13]. Pathologic CR after upfront systemic chemotherapy and short-course RT (ypCR) was specified as the absence of residual tumor cells (Mandard grade I) in primary gross tumor. Good pathologic response was defined as Mandard grade I or II (fibrosis with scattered tumor cells). We evaluated down-staging by comparing pathologic T stage to T stage at diagnosis.

### Statistical analysis

The primary endpoint of this study was progression-free survival (PFS). PFS was calculated from the date of diagnosis to the date of disease progression, relapse, death from any cause, or last follow-up. Secondary endpoints were overall actuarial survival (OS), treatment-related toxicity, and compliance. OS was calculated from the date of diagnosis to the date of death or last follow-up. PFS and OS calculation was performed by the Kaplan–Meier method using the log-rank test to estimate the statistical significance of the differences in survival. Cox’s proportional hazards model was utilized for univariate and multivariate analyses to evaluate prognostic factors that influence PFS and OS. The hazard ratio (HR) is reported with 95% confidence intervals (CIs). We compared the differences in nominal variables using Pearson’s χ^2^ test or Fisher's exact test. The continuous variables were analyzed using the Mann-Whitney U test and t test. We considered P values <0.05 significant. For all analyses, SPSS version 20.0.0 (IBM Corporation; Armonk, NY, USA) was used.

## Results

### Treatment groups based on the surgical treatment results after preoperative treatment

**Fig 1:**
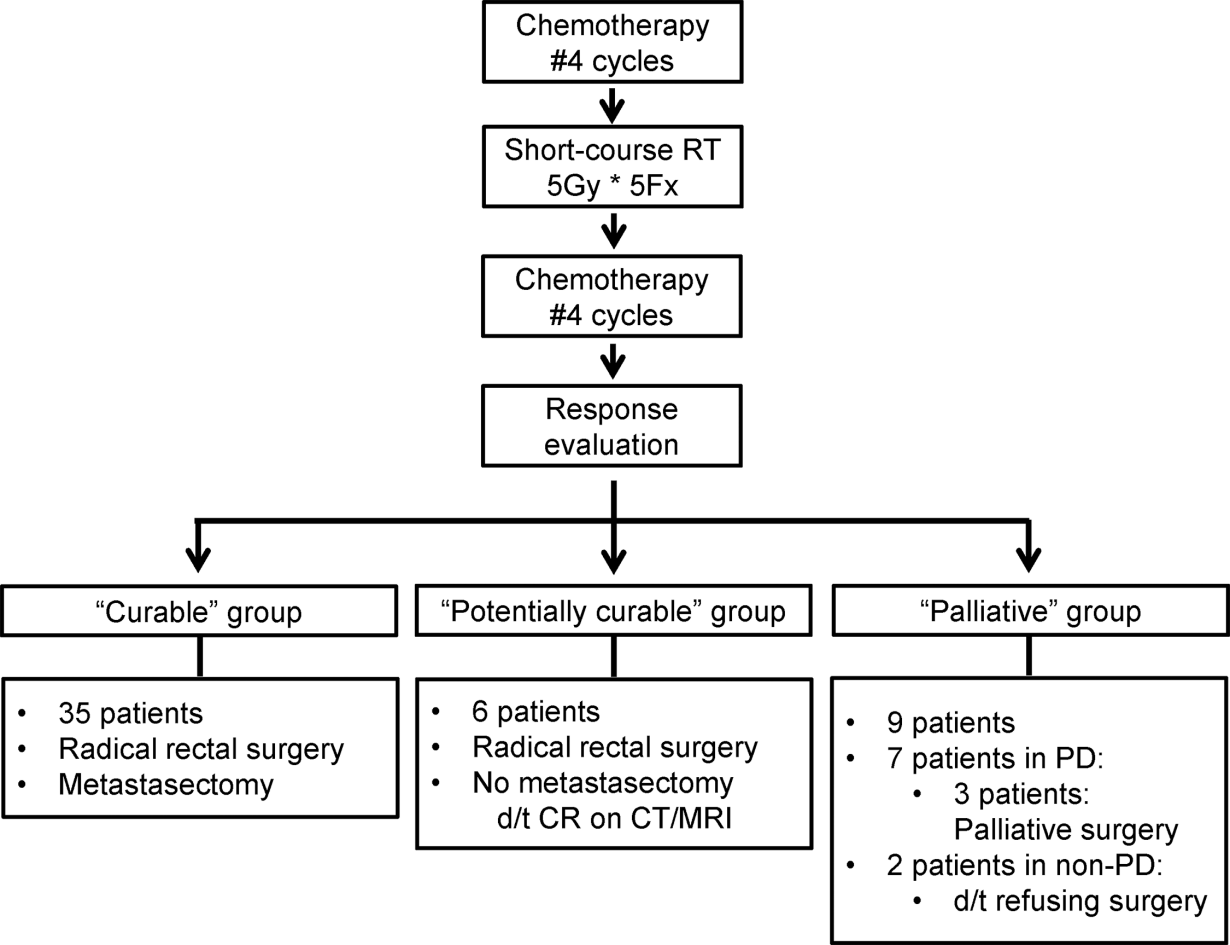
A flowchart depicting the timeline of upfront chemotherapy and short-course radiotherapy with delayed surgery at our institution.

After the completion of planned upfront chemotherapy and short-course RT, we evaluated treatment response. Fifty patients showed PR or SD for the primary tumor. Among them, PD for distant metastasis was shown in 7 patients who underwent no curative surgery. While 2 non-PD patients refused curative resection, 41 received radical surgery for their primary tumors. Among these, 35 patients (70%) also underwent metastasectomy; the remaining 6 patients (12%) showed clinical CR for distant metastasis on follow-up imaging. We categorized the 35 patients who underwent radical surgery for primary and distant metastatic tumors as the curable group; the 6 patients who received radical surgery for only the primary tumor were designated as the potentially curable group. The remaining 9 patients constituted as palliative group. There were 3 palliative group patients who received palliative surgery due to primary tumor bleeding. Hence, a total of 44 patients (88%) received rectal surgery. Low-anterior resection (LAR) was performed in 33 patients (66%), ultra-LAR with coloanal anastomosis for 8 patients (16%), and abdominoperineal resection for 3 patients (6%). A total of 33 patients (66%) had R0 status of primary and metastatic tumors after surgery. A flowchart of upfront chemotherapy and short-course RT with delayed surgery is depicted in Fig 1, and detailed treatment characteristics are shown in S1 Table.

### Patient characteristics based on the treatment groups

The patient characteristics at diagnosis are listed in Table 1. The palliative group had more poorly differentiated adenocarcinoma (P = 0.04); additionally, this group had patients showing extramural vascular invasion (P = 0.04). The potentially curable group showed more lung metastases (P = 0.006). There were no differences in the remaining characteristics among the 3 groups.

**Table 1:** Patient characteristics.

		Curable(n = 35)		Potentially curable (n = 6)		Palliative(n = 9)		
Variables	Groups	n	%	n	%	n	%	*P* value
Age (years)	<60	19	54.3%	3	50.0%	3	33.3%	0.56
	≥60	16	45.7%	3	50.0%	6	66.7%	
Sex	Male	23	65.7%	5	83.3%	6	66.7%	0.8
	Female	12	34.3%	1	16.7%	3	33.3%	
ECOG PS	0	6	17.1%	0	0.0%	0	0.0%	0.4
	1	29	82.9%	6	100.0%	9	100.0%	
Differentiation	Well	8	22.9%	0	0.0%	1	11.1%	0.04
	Moderately	24	68.6%	3	50.0%	5	55.6%	
	Poorly	2	5.7%	1	16.0%	3	33.3%	
	Unspecified	1	2.9%	2	33.3%	0	0.0%	
CEA	Median (range)	12.42 (0.85–364.1)		3.4 (0.6–164.9)		12.79 (4.18–1203.8)		0.13
K-ras status	Wild type	17	48.6%	2	33.3%	5	55.6%	0.16
	Mutant	4	11.4%	0	0.0%	3	33.3%	
	Unknown	14	40.0%	4	66.7%	1	11.1%	
Initial cT stage	cT3	28	80.0%	4	66.7%	8	88.9%	0.74
	cT4	7	20.0%	2	33.3%	1	11.1%	
Initial cN stage	cN0	1	2.9%	0	0.0%	0	0.0%	<0.99
	cN1	34	97.1%	6	100.0%	9	100.0%	
Distance from AV	Median (cm, range)	7.2 (2.7–12)		5.5 (3–10)		9 (6.5–13.4)		0.128
Mesorectal LNI	Yes	33	94.3%	6	100.0%	9	100.0%	<0.99
Lateral LNI	Yes	15	42.9%	3	50.0%	5	55.6%	0.83
EMVI	Yes	24	68.6%	3	50.0%	9	100.0%	0.04
Metastatic sites	Liver	19	54.3%	1	16.7%	5	55.6%	0.3
	Lung	8	22.9%	5	83.3%	5	55.6%	0.006
	PAN region	14	40.0%	1	16.7%	2	22.2%	0.46
	Others*	4	11.4%	2	33.3%	1	11.1%	0.35
No. of metastatic lesions	Median (range)	4 (1–17)		4 (1–13)		6 (1–21)		0.48
	≤3	17	48.6%	2	33.3%	3	33.3%	0.61
	>3	18	51.4%	4	66.7%	6	66.7%	
Distant metastasis	Solitary	24	68.6%	3	50.0%	4	44.4%	0.35
	Multiple	11	31.4%	3	50.0%	5	55.6%	

*: Ovary, axillary & supraclavicular lymphatics

Abbreviation: ECOG PS, Eastern Cooperative Oncology Group performance status; CEA, Carcinoembryonic antigen; AV, anal verge; LNI, lymph node involvement; EMVI, extramural vascular invasion; PAN, para-aortic nodal.

### Pathologic reports for primary tumor, residual tumor status, and patterns of failure

Among 44 patients who underwent surgery for rectal lesions, Mandard grade I (ypCR) was shown in 6 patients (13.6%), II in 13 patients (29.6%), III in 16 patients (36.3%), IV in 7 patients (15.9%), and V in 2 patients (4.6%). Good pathologic response and down-staging were observed in 19 (42.3%) patients and 20 (45.5%) patients, respectively. The pathology reports are listed in Table 2. The surgical pathology characteristics, including Mandard grade I–II, ypCR, down-staging, lymphovascular invasion, perineural invasion, and positive resection margins were not different among the 3 groups (Table 3). Curable and potentially curable groups achieved an R0 status of 80% and 83.3%, respectively. None of the palliative group patients achieved an R0 status (P<0.001). Although local and regional failures were not different among the three groups, more distant failure tended to occur in the palliative group (88.9%; P = 0.07).

**Table 2:** Pathology reports (n = 44).

Variables	Groups	n	%
Mandard grade for primary tumor	I	6	13.6%
	II	13	29.6%
	III	16	36.3%
	IV	7	15.9%
	V	2	4.6%
ypT stage	ypT0	6	13.6%
	ypT1	1	2.3%
	ypT2	5	11.3%
	ypT3	30	68.2%
	ypT4	2	4.6%
ypN stage	ypN0	20	45.5%
	ypN1	10	22.7%
	ypN2	14	31.8%
Down-staging	Yes	20	45.5%
	No	30	54.5%
Lymphovascular invasion	Yes	9	20.5%
Perineural invasion	Yes	3	6.8%

**Table 3:** Surgical pathology for primary tumor, residual tumor status, and patterns of failure based on treatment groups.

	Curable	Potentially curable	Palliative	
	n (%)	n (%)	n (%)	*P* value
Surgical pathology* (n = 44)				
• Mandard grade I-II	15 (42.9)	3 (50.0)	1 (33.3)	<0.99
• ypCR	5 (14.3)	1 (16.7)	(0.0)	1
• Downstaging	14 (40.0)	5 (83.3)	1 (33.3)	0.118
• Lymphovascular invasion	8 (22.9)	0 (0.0)	1 (33.3)	0.17
• Perineural invasion	3 (8.6)	0 (0.0)	0 (0.0)	0.65
• Resection margin (+)	3 (8.6)	1 (16.7)	0 (0.0)	0.61
Residual tumor status				
• R0	28 (80.0)	5 (83.3)	0 (0.0)	<0.001
• R1 or R2	7 (20.0)	1 (16.7)	9 (100.0)	
Patterns of failure				
• Local failure	2 (5.7)	0 (0.0)	0 (0.0)	<0.99
• Regional failure	1 (2.9)	0 (0.0)	0 (0.0)	<0.99
• Distant failure	18 (51.4)	2 (33.3)	8 (88.9)	0.07

*: except 6 patients who underwent no rectal surgery

Abbreviation: ypCR, pathologic complete response after preoperative treatment.

### Survival analysis and favorable prognostic factors

**Fig 2:**
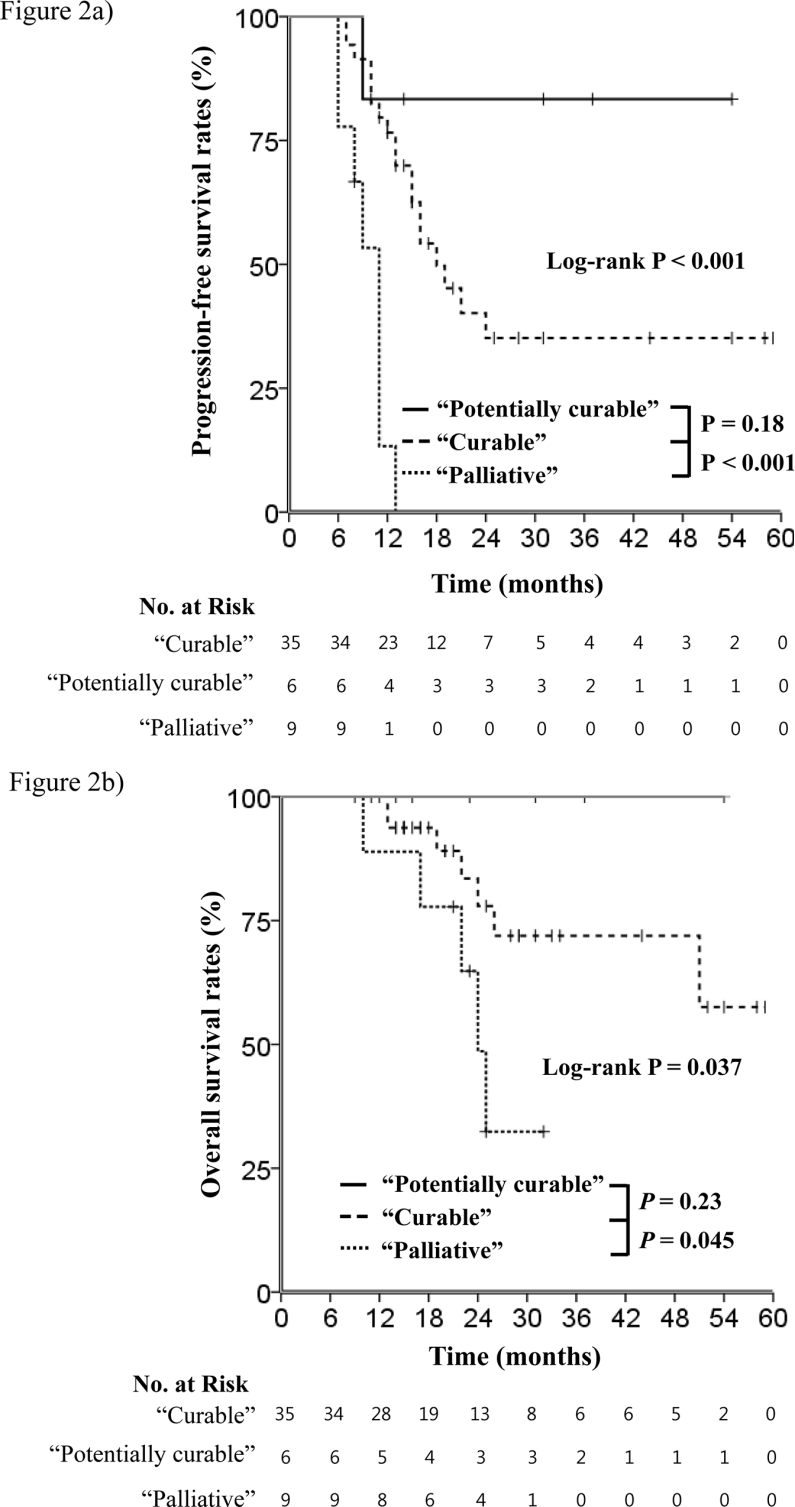
Kaplan-Meier curves according to treatment groups. (a) The progression-free survival rates of the curable (broken line) and potentially curable (line) groups were significantly longer than that of the palliative group (dotted line). (b) The curable (broken line) and potentially curable (line) groups showed longer overall survival than the palliative group (dotted line).

Median follow-up duration for surviving patients was 22 months (range, 9–59 months). Median PFS was 16 months, and the 2-year PFS rate was 34.8%. The 2- and 5-year OS rates were 73.9% and 55.1%, respectively. Kaplan-Meier curves demonstrated that PFS rates of the curable and potentially curable groups were significantly longer than that of the palliative group (Fig 2A, curable vs. potentially curable vs. palliative groups, median PFS 18 months vs. not reached vs. 11 months, P<0.001). Kaplan-Meier analysis also showed that the curable and potentially curable groups showed longer OS than the palliative group (Fig 2B, curable vs. potentially curable vs. palliative groups, median OS not reached vs. not reached vs. 24 months, P = 0.037).

On univariate analysis of PFS with clinical prognostic factors, the number of metastatic lesions (≤3 vs. >3), pattern of distant metastasis (solitary vs. multiple), residual tumor status (R0 vs. R1–2), and good pathologic response in primary tumor (yes vs. no) were significantly associated with PFS (Table 4). On multivariate analysis, the pattern of distant metastasis (solitary vs. multiple) and residual tumor status (R0 vs. R1–2) were independent prognostic factors that influenced PFS significantly. The number of metastatic lesions affected PFS with a statistical trend (P = 0.057). Residual tumor status (P = 0.002) and carcinoembryonic antigen (P = 0.005) were significantly related to OS on univariate analysis (Table 4). The residual tumor status (R0 vs. R1–2: HR, 0.12; 95% CI, 0.03–0.6; P = 0.01) was an independent significant prognostic factor for OS.

**Table 4:** Stepwise uni- and multi-variate analysis using cox regression model for progression-free and overall survival.

	Progression-free survival			Overall survival		
	Univariables	Multivariable		Univariables	Multivariable	
Variables	*p* value	HR (95% CI)	*p* value	*p* value	HR (95% CI)	*p* value
Age (continuous)	0.47			0.48		
Gender (male vs. female)	1			0.88		
ECOG performance (0 vs. 1)	0.23			0.52		
Differentiation (others vs. poorly differentiated)	0.95			0.19		
CEA (continuous)	0.11			0.005	1.002 (1–1.004)	0.03
K-ras mutation (others vs. K-ras mutation)	0.99			0.33		
Clinical T stage (T3 vs. T4)	0.43			0.92		
Distance from anal verge (cm) (continuous)	0.45			0.39		
Extramural vascular invasion (no vs. yes)	0.27			0.51		
Liver metastasis (no vs. yes)	0.26			0.7		
Lung metastasis (no vs. yes)	0.61			0.88		
PALN metastasis (no vs. yes)	0.51			0.48		
No. of metastatic lesion (≤3 vs. >3)	0.006	0.42 (0.18–1.03)	0.057	0.34		
Pattern of distant metastasis (solitary vs. multiple)	0.002	0.34 (0.15–0.75)	0.008	0.22		
Residual tumor status (R0 vs. R1-2)	0.001	0.24 (0.1–0.56)	0.001	0.002	0.12 (0.03–0.6)	0.01
Good pathologic response in PT (Yes vs. no)	0.03			0.09		
Downstaging (Yes vs. no)	0.32			0.19		

Abbreviation: ECOG, Eastern Cooperative Oncology Group; CEA, Carcinoembryonic antigen; PALN, para-aortic lymph node; PT, primary tumor; HR, hazard ratio; CI, confidence interval.

### Treatment-related toxicity and the compliance

Treatment-related toxicities are shown in Table 5. Upfront chemotherapy-related severe hematologic and gastro-intestinal toxicities developed in 3 (6%) and 4 (8%) patients, respectively. Upfront chemotherapy and short-course RT-related grade 3 diarrhea developed acutely in 17 patients (35%). However, after the completion of treatment, diarrhea resolved in all patients. As for surgical complications, anastomotic leakage was observed in 7 patients (16%); 3 major and 4 minor. Perineal infection occurred in 2 patients (5%). Upfront chemotherapy and short-course RT with delayed surgery was considered tolerable.

**Table 5:** Treatment-related toxicity.

	n	%
Chemotherapy-related grage 3 toxicity		
• Hematologic	3	6%
• GI (nausea/vomiting/mucositis)	4	8%
• Neuropathy	0	0%
• Gerenal weakness	0	0%
Chemotherapy and SCRT-related grage 3 toxicity		
• Diarrhea	17	35%
Surgery-related toxicity		
• Anastomotic leakage	7	16%
Major	3	7%
Primary repair & I/D	3	7%
Loop ileostomy	2	5%
Minor	4	9%
Transanal reinforcement suture	2	5%
Pigtail insertion	1	2%
Observation (Asymptomatic)	1	2%
• Perineal infection	2	5%
• Chyle leakage	1	2%

Abbreviation: SCRT, short-course radiotherapy.

Median treatment duration from diagnosis to surgery was 5.4 months (range, 1.7–9.0 months). Median time between completion of short-course RT and surgery was 3.3 months (range, 0.3–4.4 months). Median hospitalization duration for patients who underwent surgery was 11 days (range 6–91 days).

## Discussion

While systemic combination chemotherapy is critical for rectal cancer with distant metastasis, long-course RT is considered the appropriate treatment for LARC according to NCCN guideline [14]. However, long-course RT for LARC with distant metastasis has some problems as follows: 1) the start of systemic combination chemotherapy is delayed; 2) long-course RT could not be performed concurrently with systemic combination chemotherapy due to treatment-related acute toxicity. Recently, some studies including T4 patients demonstrated that short-course RT followed by delayed surgery could improve local control rates for LARC patients [8,9,15]. So, we choose short-course RT and systemic combination chemotherapy to control both local tumor and distant metastasis without severe acute toxicities.

The effect of systemic chemotherapy and short-course RT followed by delayed surgery for rectal cancer diagnosed with distant metastasis has not been well established. A Dutch group demonstrated that short-course RT and chemotherapy followed by surgery could be attractive alternatives to traditional staged resection for rectal cancer with synchronous liver metastases, based on the achievement of 15 months of disease-free survival, a 5-year OS of 38%, and tolerability of treatment [16]. An open-label, single-arm phase II prospective clinical study recently reported that short-course RT followed by combination chemotherapy including capecitabine and oxaliplatin with bevacizumab and subsequent radical surgery could be a potentially curative treatment and was feasible for 50 rectal cancer patients of similar severity [10]. The rate of R0 status, which was the primary endpoint of their study, was 72%; the 2-year OS and recurrence rate were 80% and 64%, respectively. Treatment-related deaths and grade 3–4 short-course RT-induced toxicities did not develop. Our findings were comparable to those of previous studies, and we deduced that upfront systemic chemotherapy and short-course RT followed by delayed surgery is an effective and feasible treatment for LARC with potentially resectable distant metastasis.

While long-course CCRT followed by delayed surgery can induce tumor down-staging, short-course RT followed by immediate surgery is not known to be effective for tumor regression despite no statistical difference of survival outcomes compared to long-course CCRT followed by delayed surgery [17,18]. Several studies demonstrated that short-course RT followed by delayed surgery could result in substantial tumor regression and down-staging for LARC patients [8,9,15]. However, other studies insisted that short-course RT followed by delayed surgery did not increase tumor down-staging and showed poor pathologic responses compared to long-course conventional CCRT [19,20]. The only prospective study of short-course RT followed by delayed surgery for rectal cancer diagnosed with distant metastasis demonstrated that patients had a ypCR rate of 26% and a down-staging rate of 47% [10]. Consistent with previous studies, we showed a ypCR rate of 13.6% and a down-staging rate of 45.5%. We propose that short-course RT with delayed surgery will induce substantial down-staging for patients with MRF involvement.

Systemic chemotherapy has been a critical treatment for metastatic rectal cancer to manage metastatic lesions and eradicate microscopic disease [14]. Several studies demonstrated that systemic chemotherapy could help improve outcomes [21,22]. These studies also showed that pathologic response to neoadjuvant chemotherapy would predict survival of patients with resectable metastases from colorectal cancer [23,24]. In our study, 6 patients in the potentially curable group showed clinical CR of distant metastasis after systemic chemotherapy alone, resulting in an excellent survival outcome. Taken together, cumulative data confirm that systemic chemotherapy is an essential treatment for enhancing survival rates of LARC patients with synchronous metastatic disease.

There is great heterogeneity in rectal cancer patients diagnosed with distant metastases, which can complicate treatment decisions. Because there are no clear criteria for selecting patients to whom to apply this treatment strategy, it is important to elucidate the clinical factors that can identify patients who would benefit from it. Based on previous studies, one of the most consistently reported factors is multiplicity of metastases. Many other studies demonstrated that solitary or low numbers of metastasis showed a significantly better survival rate than multiple metastases of the liver, lung, and other organs [25–30]. Our study also indicated that multiplicity of metastasis was significantly associated with PFS. Therefore, we propose that this treatment scheme should enhance survival for colorectal cancer patients with fewer metastatic lesion or a solitary metastatic organ.

In our study, there were 9 patients (palliative group) who did not undergo surgery after preoperative treatments. While 3 patients were treated with palliative surgery because of severe tumor bleeding, the remaining 6 patients had no severe pelvic symptoms including rectal bleeding, pain, and obstruction. Recently, one prospective study described findings similar to ours [31]. Of 40 patients, only 8 (20%) underwent palliative surgery because of local symptomatic progression. Although all patients had severe pelvic symptoms before RT, pelvic symptoms were completely resolved during the whole course of the disease in 30% of patients, and significant symptomatic improvement was observed in 35% of patients. Our results suggest that upfront systemic chemotherapy and short-course RT could help relieve pelvic symptoms caused by rectal cancer.

There were several limitations to consider. First, since this study is retrospective, patient characteristics were heterogeneous; furthermore, targeted therapeutic drugs were not administered to 38 patients (76%). Especially, this study included various sites of metastasis. The heterogeneity of metastatic organ could induce the different effects of this treatments strategy on the prognosis, despite multivariate analysis including several factors related to metastasis. So, further study involving the large number of homogenous patients would be needed. Second, the number of patients in this study was small. Third, there are controversies regarding the definition of resectability for metastatic lesions. At our institution, resectability was determined based on the extent of metastatic disease and functional reserve of the non-metastatic organ region. Fourth, because all of the stage IV LARC patients with limited number of metastatic lesions between 2009 and 2014 underwent upfront systemic chemotherapy and short-course RT with delayed surgery, we could not compare the chemotherapy only, or chemotherapy and long course RT with the treatment scheme of this study. This randomization study would be warranted to confirm role of pelvic radiotherapy in metastatic setting. So, further study would be needed. Despite these limitations, we consider our findings reliable.

## Conclusions

In conclusion, our data demonstrate a strong possibility that upfront chemotherapy and short-course RT with delayed surgery are an effective alternative treatment for LARC with potentially resectable distant metastasis, owing to achievement of pathologic down-staging, R0 resection, and favorable compliance and toxicity, despite the long treatment duration.

## Supporting Information

S1 Table Treatment characteristics. The detailed treatment characteristics are shown.
(DOCX)
