# Supplementary material for: Upfront Systemic Chemotherapy and Short-Course Radiotherapy with Delayed Surgery for Locally Advanced Rectal Cancer with Distant Metastases: Outcomes, Compliance, and Favorable Prognostic Factors
Source: PLoS One. 2016 Aug 18;11(8):e0161475. doi: 10.1371/journal.pone.0161475 (PMC4990310; doi:10.1371/journal.pone.0161475)
Supplement: S1 Table — The detailed treatment characteristics are shown. (DOCX) [file pone.0161475.s001.docx]

Supplementary table 1 Treatment characteristics

| Variables | Groups | n | % |
| --- | --- | --- | --- |
| Chemotherapy regimen | FOLFOX | 43 | 86% |
|  | FOLFIRI | 6 | 12% |
|  | Capcitabine mono | 1 | 2% |
| No of cycle before SCRT | Median (range) | 4 (4-9) | |
| Total dose (Gy) |  | 25 | |
| Fraction size (Gy) |  | 5 | |
| Total cycle of chemotherapy | Median (range) | 8 (4-12) | |
| Targeted therapy | None | 38 | 78% |
|  | Bevacizumab | 9 | 18% |
|  | Cetuximab | 2 | 4% |
| The type of surgery for primary tumor | Low anterior resection (LAR) | 33 | 66% |
|  | Ultra-LAR c coloanal anastomosis | 8 | 16% |
|  | Abdominoperineal resection | 3 | 6% |

Abbreviation: SCRT, short-course radiotherapy; FOLFOX, folinic acid (leucovorin), fluorouracil, oxaliplatin; FOLFIRI, folinic acid (leucovorin), irinotecan
